# Engaging men in HIV programmes: a qualitative study of male engagement in community‐based antiretroviral refill groups in Zimbabwe

**DOI:** 10.1002/jia2.25403

**Published:** 2019-11-05

**Authors:** Joanne E Mantell, Tsitsi B Masvawure, Munyaradzi Mapingure, Tsitsi Apollo, Clorata Gwanzura, Laura Block, Eleanor Bennett, Peter Preko, Godfrey Musuka, Miriam Rabkin

**Affiliations:** ^1^ Division of Gender Sexuality and Health Department of Psychiatry Columbia University New York State Psychiatric Institute New York NY USA; ^2^ Center for Interdisciplinary Studies College of the Holy Cross Worcester MA USA; ^3^ ICAP at Columbia University Harare Zimbabwe; ^4^ HIV/AIDS and STIs Unit Ministry of Health and Child Care Harare Zimbabwe; ^5^ ICAP at Columbia University New York NY USA; ^6^ ICAP at Columbia University Mbabane Eswatini; ^7^ Departments of Medicine & Epidemiology Columbia University Mailman School of Public Health New York NY USA

**Keywords:** HIV, male engagement, differentiated service delivery, community antiretroviral groups, Zimbabwe, qualitative

## Abstract

**Introduction:**

Suboptimal male engagement in HIV programmes is a persistent challenge, leading to lower coverage of HIV testing, prevention and treatment services, and to worse outcomes for men. Differentiated service delivery models, such as peer‐led community antiretroviral refill groups (CARGs), offer the opportunity to enhance patient satisfaction, retention and treatment outcomes. We conducted an exploratory qualitative study to identify facilitators and barriers to CARG participation by HIV‐positive men, with inputs from recipients of HIV care, community members, healthcare workers (HCWs), donors and policymakers.

**Methods:**

Between July and October 2017, we conducted 20 focus group discussions (FGDs) with 147 adults living with HIV, including men and women enrolled in CARGs and men not enrolled in CARGs, and 46 key informant interviews (KIIs) with policymakers, donors, HCWs and community members. FGDs and KIIs were recorded, transcribed and translated. A constant comparison approach was used to triangulate findings and identify themes related to male engagement in CARGs in rural Zimbabwe.

**Results:**

CARG participants, policymakers, donors, HCWs, and community members noted many advantages to CARG participation, including convenience, efficiency, solidarity and mutual psychosocial support. Although those familiar with CARGs reported that these groups decreased HIV‐related stigma, concerns about stigma and privacy were perceived to be the primary reason for men’s non‐participation. Other important barriers to male enrolment included lack of awareness of CARGs, misunderstanding of how CARGs operate, few perceived benefits and lack of flexibility in CARG implementation.

**Conclusions:**

More effective educational and awareness campaigns, community‐based anti‐stigma campaigns, more flexible CARG designs, and provision of financial and/or in‐kind support to CARG members could mitigate many of the barriers to male enrolment in CARGs. Men may also prefer alternative differentiated service delivery models that are facility‐based and/or do not require group participation.

## 1 Introduction

Engaging men in HIV testing, prevention and treatment is a persistent challenge 1, 2, 3, 4. In sub‐Saharan Africa (SSA), population‐based HIV surveys show that men consistently lag behind women in awareness of their HIV status 5. In some settings, men diagnosed with HIV are also less likely to link to care, adhere to antiretroviral therapy (ART) and maintain viral suppression 6, 7, 8, 9. Although women and girls are disproportionately affected by HIV, the effective engagement of men and boys is also critical for both equity and effective HIV epidemic control.

Suboptimal male engagement in HIV programmes is typically attributed to several key barriers. Men interact with the health system less frequently than women, who are more likely to visit health facilities (HFs) in the context of family planning, antenatal services and paediatric care 10. These contacts provide ongoing opportunities to access information about HIV and related services, including opt‐out HIV testing which is offered on a routine basis in these settings. Male gender norms present a second barrier, prompting concerns about compromised masculinity for men who use health services in general and HIV services in particular 11, 12, 13, 14. Stigma is another important deterrent of male engagement with HIV services 15, 16.

**Figure 1:**
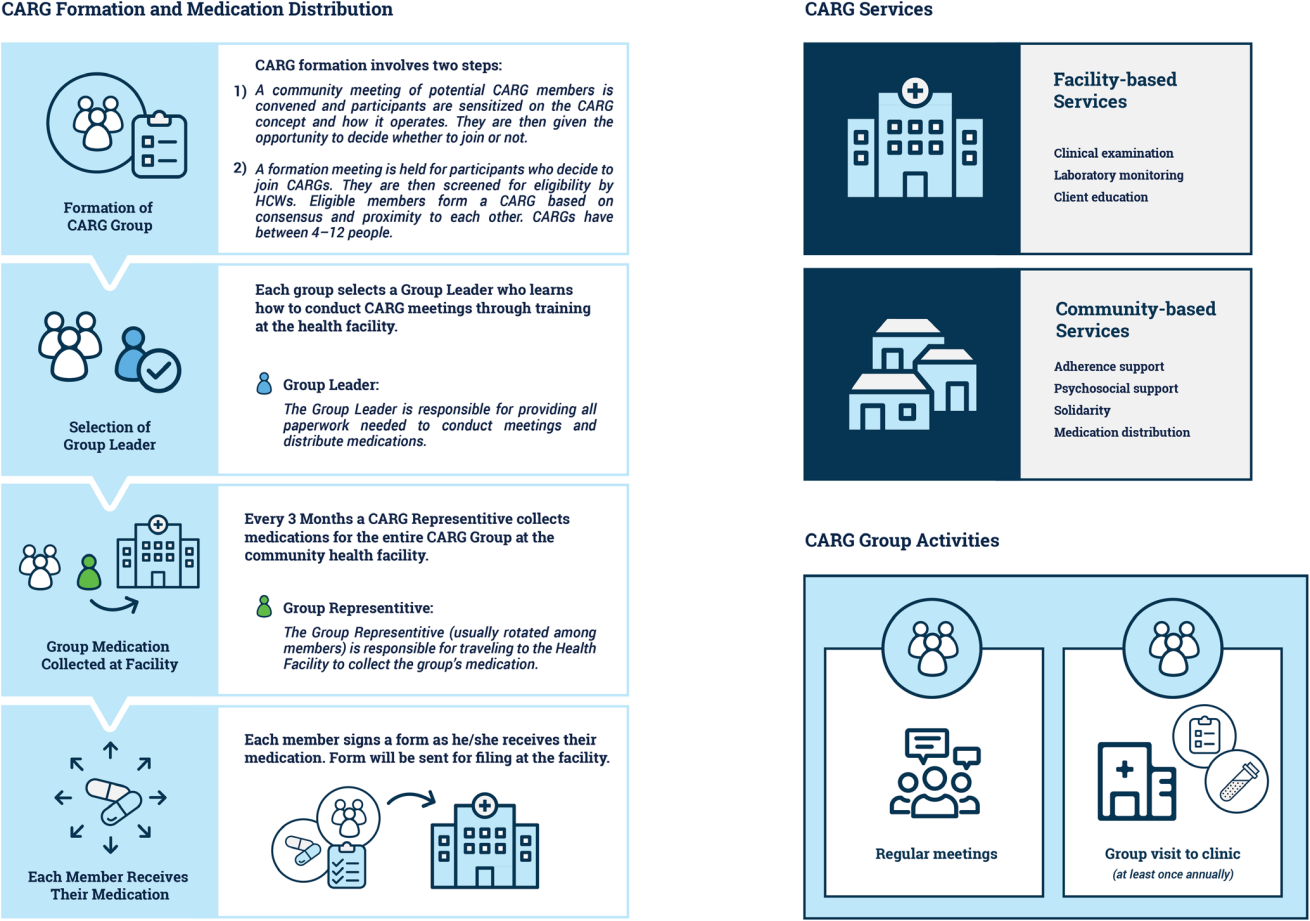
Overview of CARGs.

HIV differentiated service delivery models (DSDM) tailor HIV‐related services to the needs and preferences of subsets of persons living with HIV and may mitigate some of the barriers to male engagement 17, 18. One successful DSDM is the community‐based, patient‐led group treatment model, broadly known as Community Antiretroviral Groups (CAGs) 19, and in Zimbabwe as Community Antiretroviral Refill Groups (CARGs) 20. CAGs have been widely implemented, and early evidence suggests that participants who opt‐in voluntarily do as well or better than their peers in conventional HF‐based care 21, 22, 23. In Zimbabwe, stable patients doing well on ART are eligible to join CARGs, which offer solidarity, psychosocial support and community‐based medication refills while reducing HF visit frequency (Figure 1).

In 2016, 68% of Zimbabwean men were aware of their HIV status versus 76% of women; 59% of men aware of their HIV status were on ART versus 66% of women; and 84% of men on ART were virologically suppressed versus 88% of women 24. Although CARGs were not specifically designed to increase male engagement, Zimbabwe’s Ministry of Health and Child Care (MoHCC) and its partners hope that the model may encourage more men to remain in HIV care and achieve viral suppression. We conducted an exploratory qualitative study to identify facilitators and barriers to CARG participation by HIV‐positive men, with inputs from recipients of HIV care, community members, healthcare workers (HCWs), donors and policymakers.

## 2 Methods

### 2.1 Study setting

In consultation with MoHCC, we purposively selected three clinics with active CARGs in two rural districts in Zimbabwe’s Mashonaland Central and Mashonaland West Provinces (Table 1).

**Table 1:** CARG characteristics at study sites

Introduction of CARGs			
Health Facility 1	2014		
Health Facility 2	2014		
Health Facility 3	2017		
Number of patients on ART (as of December 2017)			
	Total (100%)	< 15 years	15 + years
Health Facility 1	1145	91 (7.9%)	1054 (92.1%)
Health Facility 2	1155	90 (7.8%)	1065 (92.2%)
Health Facility 3	231	2 (0.9%)	229 (99.1%)
Proportion of patients in CARGs since launch			
Health Facility 1	9.3% (107/1145)		
Health Facility 2	8.7% (100/1155)		
Health Facility 3	8.2% (19/231)		
Proportion of patients 15 + years in CARGs by sex			
Health Facility 1	74% (79/107) female		
	26% (28/107) male		
Health Facility 2	58% (58/100) female		
	42% (42/100) male		
Health Facility 3	68% (13/19) female		
	32% (6/19) male		

### 2.2 Study design, sampling and data collection

Between July and October 2017, we conducted 20 focus group discussions (FGDs) and 46 key informant interviews (KIIs). Participants were purposively selected to obtain diverse perspectives. FGDs were conducted with three groups of HIV‐positive individuals (N = 147): men enrolled in CARGs (four with men 18 to 35 years and four with men >35 years); men eligible for but not enrolled in CARGs (four with men 18 to 35 years and four with men >35 years); and women enrolled in CARGs (four). KII participants included: (1) central‐level informants from MoHCC, donors, programme implementers, community‐based organizations and organizations of people living with HIV; (2) HF‐level informants (clinicians, peer educators, counsellors); and (3) community‐level informants (community and religious leaders, community health workers).

FGD and IDI guides were developed in English, translated into Shona, back‐translated into English, revised and piloted for clarity. FGDs were conducted in Shona and KIIs in English by bilingual Zimbabwean research assistants. FGDs were facilitated by two researchers of the same sex as the participants. FGDs and KIIs were audio‐recorded, transcribed and translated into English by the researcher who facilitated that group’s discussion or interview. Local research assistants participated in a week‐long training workshop on qualitative data collection techniques and had daily de‐briefings with a trained supervisor as well as ongoing supervision.

### 2.3 Data analysis

Data were coded and analysed using an iterative process. An initial codebook was developed deductively from the interview guide and then inductively from transcripts. Two researchers, working independently, read the same transcripts and identified key themes using thematic analysis 25. They compared their themes and also incorporated *a priori* themes based on the interview guide to develop the initial codebook. This codebook was entered into Dedoose™, a qualitative data analysis software program, and the two researchers independently coded two transcripts each, compared their coding and further refined the codebook. All transcripts were then double‐coded by the two researchers, who met regularly to compare and reach consensus. Data analysis was based on a constant comparison approach 26, 27, 28 of major themes and emergent categories including: “reasons for not joining CARGs,” “advantages/benefits of CARGs,” “challenges of CARGs and “recommendations to increase male engagement.” This iterative approach involved constantly comparing identified themes to assess their scope, patterns, relationships, and conceptual similarities and differences.

Analysis of code reports was a collaborative iterative effort, allowing for different data interpretations to be explored and further analysis of transcripts to be conducted until consensus was achieved, thus minimizing potential biases in interpretation.

### 2.4 Ethical considerations

The MoHCC, Columbia University Medical Center Institutional Review Board (Protocol IRB‐AAAR4364), and the Medical Research Council of Zimbabwe (Protocol MRCZ/A/2092) approved the study protocol.

All participants completed written informed consent and were assured that they could decline to answer questions and/or withdraw from the study at any point. No personal identifiers were collected. FGD participants were informed about the group nature of the discussions and that while confidentiality could not be assured, all FGD participants had been asked not to talk about other’s contributions outside of the group.

## 3 Results

**Figure 2:**
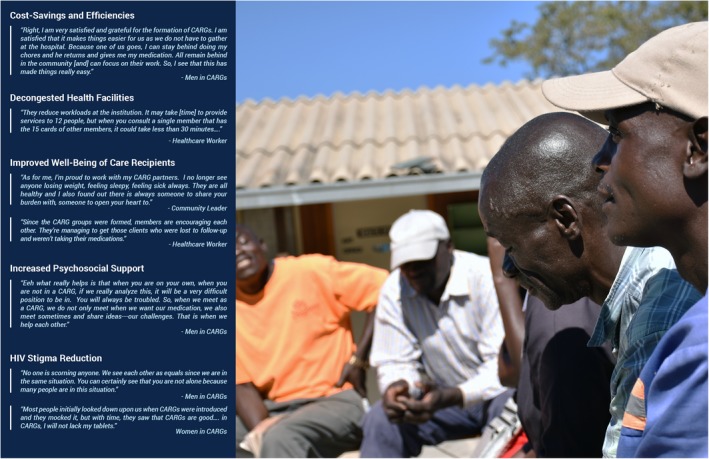
Benefits of CARGs.

**Figure 3:**
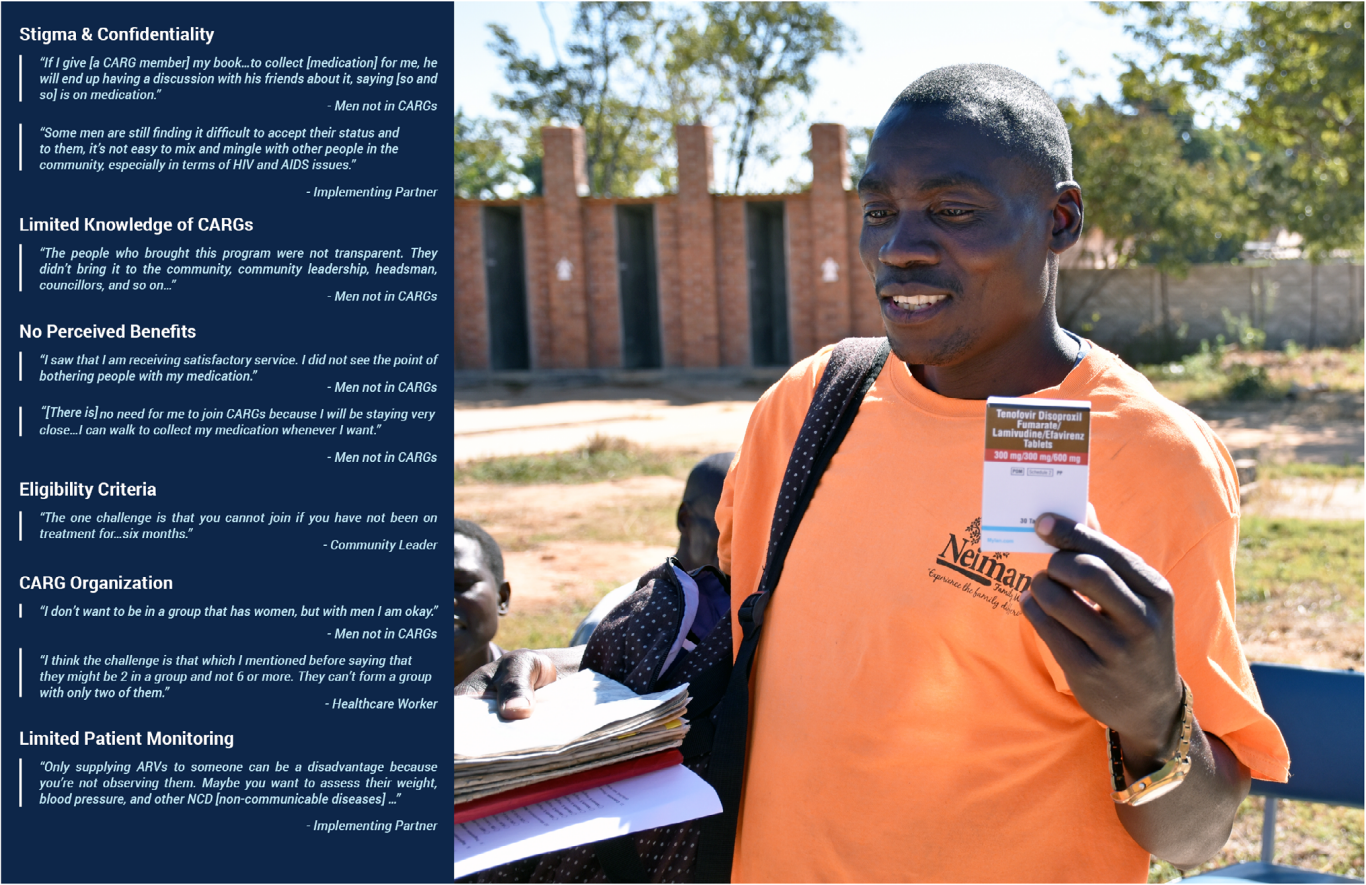
Barriers to Male Participation in CARGs.

Table 2 describes our sample. Of note, most community members were self‐employed or employed part‐time, and many reported earning less than USD100 in the month preceding the study. Many community members also had partners who were HIV positive. Additionally, most HCWs and central‐level participants had been working at their organizations for 2 and 3 years, respectively. Table 3 shows the distribution of themes by participant type. Figures 2 and 3 highlight illustrative quotes from study participants. There were no substantive differences between the responses from younger versus older men in FGDs.

**Table 2:** Sociodemographic characteristics of participants (N = 198)

	Men on ART Enrolled in CARG N = 57		Men on ART Not Enrolled in CARG N = 61		Women on ART Enrolled in CARG N = 29	Community Leaders N = 16	Health Care Providers N = 15	MoHCC, Programme implementers NGO, Religious Organizations N = 15 (20 participants)^a^
Age group	18 to 35 years n = 28 (%)	36 + years. n = 29 (%)	18 to 35 years n = 29	36 + years n = 32	>18 years	–	–	–
Characteristics^b^	n (%)	n (%)	n (%)	n (%)	n (%)	n (%)	n (%)	n (%)
Male	28 (100)	29 (100)	29 (100)	32 (100)		9 (56)	5 (33)	14 (70)
Women					29 (100)	7 (44)	10 (67)	6 (30)
Age						45 (40.5 to 59)	36 (32 to 47)	35 (32 to 39)
Median (IQR)	30.5 (29 to 33.5)	47 (42 to 51)	30 (25 to 33)	46 (41 to 55.5)	47 (41 to 57)			
Marital status						–	–	–
Single	6 (21)	0 (0)	7 (24)	0 (0)	0 (0)			
Married monogamous	16 (57)	22 (76)	10 (34)	19 (59)	10 (34)			
Married polygamous	0 (0)	0 (0)	4 (14)	0 (0)	3 (10)			
Cohabiting	3 (11)	1 (3)	6 (21)	3 (9)	1 (3)			
Divorced	2 (7)	1 (3)	2 (7)	5 (16)	4 (14)			
Separated	1 (4)	2 (8)	0 (0)	1 (3)	0 (0)			
Widowed	0 (0)	3 (10)	0 (0)	4 (13)	11 (38)			
Highest educational level						–	–	–
None	2 (7)	1 (3)	1 (4)	2 (6)	3 (10)			
Primary	6 (21)	5 (17)	1 (4)	11 (34)	18 (62)			
Secondary	19 (68)	21 (73)	26 (88)	18 (56)	8 (28)			
Tertiary	1 (4)	2 (7)	1 (4)	1 (3)	0 (0)			
Religion						–	–	–
Apostolic	9 (32)	7 (24)	9 (31)	7 (22)	13 (45)			
Christian	8 (29)	8 (28)	7 (24)	14 (44)	8 (28)			
Protestant	1 (4)	3 (10)	1 (3)	1 (3)	7 (24)			
Traditional	3 (11)	5 (17)	2 (7)	2 (6)	0 (0)			
Muslim	0 (0)	0 (0)	2 (7)	1 (3)	0 (0)			
None	7 (25)	6 (21)	8 (28)	7 (21)	1 (3)			
How earn money						–	–	–
Full time employed	5 (18)	0 (0)	1 (3)	0 (0)	0 (0)			
Part time employed	6 (21)	2 (7)	4 (14)	15 (47)	0 (0)			
Informally employed	2 (7)	7 (24)	1 (3)	4 (13)	0 (0)			
Temporary jobs	5 (18)	3 (10)	3 (10)	1 (3)	0 (0)			
Self employed	6 (21)	11 (38)	14 (48)	9 (28)	15 (52)			
Other	0 (0)	0 (0)	3 (10)	0 (0)	9 (31)			
Not earning any money	4 (14)	6 (21)	3 (10)	3 (9)	5 (17)			
Income earned last month						–	–	–
≤$100	16 (57)	25 (86)	15 (52)	25 (78)	28 (97)			
$101 to $500	6 (21)	1 (3)	1 (3)	6 (19)	1 (3)			
Don’t know	3 (11)	3 (10)	7 (24)	1 (3)	0 (0)			
No answer	3 (11)	0 (0)	6 (21)	0 (0)	0 (0)			
Partner HIV status						–	–	–
HIV positive	17 (61)	18 (62)	11 (38)	20 (63)	13 (45)			
HIV negative	4 (14)	6 (21)	10 (34)	3 (9)	5 (17)			
Unknown	2 (7)	0 (0)	4 (14)	6 (19)	1 (3)			
N/A	2 (7)	2 (7)	0 (0)	0 (0)	0 (0)			
Refused to answer	3 (11)	3 (10)	4 (14)	3 (9)	10 (34)			
Length of time living in community	>12 months	>12 months	>12 months	>12 months	>12 months	29 (22 to 40)	–	–
Length of time in community leader role	–	–	–	–	–	3.5 (2 to 6.5)	–	–
Position/role	–	–	–	–	–	c	d	e
Involved in direct clinical care	–	–	–	–	–			
Yes						0 (0)	12 (80)	11 (55)
No						15 (100)	3 (20)	6 (30)
Not stated						0 (0)	0 (0)	3 (15)
Length of time working at facility/organization	–	–	–	–	–	–	2 (0.5 to 7)	2.7 (1.2 to 8)
Organization type	–	–	–	–	–	–	Health Care Facility	f

a In some organizations, the leaders invited more than one person to the interview;

b Median and IQR are reported for continuous variables;

c Children Care worker, 4 Village Health workers, Head lady, Chief, Health committee Chairman, District CARGs Focal Person, 2 Ward Councillors, CARG Leader, CARG focal person, Community Linkage Facilitator, Village Development Committee Chairperson, Community Chairperson;

d 2 Sisters in charge, 7 Registered General Nurses, Primary Counsellor, Pharmacy Manager, 2 Primary Care Nurses, Microscopist, Community Linkage Facilitator;

e Health Promotion Officer, 2 District Medical Officers, District Nursing Officer, Medical Doctor, District Community Linkages Officer, Country Medical Coordinator, Volunteer, Program Officer, Acting CEO, 2 HIV Technical Advisors, National Coordinator, Clinical Advisor, National ART Coordinator, Public Health Specialist, Regional HIV Coordinator, Mentor, Intern, District AIDS Coordinator;

f *Médecins Sans Frontières,* Ministry of Health and Child Care, International Training and Education Centre for Health (I‐TECH), Zimbabwe National Network for People Living with HIV, FHI360, USAID, National AIDS Council, US Centers for Disease Control and Prevention Zimbabwe, Zvandiri.

**Table 3:** Participants’ views on CARG benefits, barriers and recommendations for increasing male engagement

	Participant Group					
	FGDs			IDIs		
	*MoC* *N = 8*	*MinC* *N = 8*	*WinC* *N = 4*	*CoL* *N = 15*	*HCW* *N = 16*	*CeL* *N = 15*
CARG benefits						
Advantages of CARGs						
Reduced stigma	2/8	3/8	3/4	5/13	3/16	2/11
Convenience (time and cost savings)	5/8	8/8	3/4	6/13	13/16	9/11
Psycho‐social support	5/8	5/8	4/4	3/13	4/16	6/11
Improved patient health	2/8	5/8	1/4	4/13	2/16	4/11
Decongested facilities	0/8	2/8	2/4	5/13	14/16	7/11
CARG barriers						
Men’s reasons for not joining CARGs						
Privacy/stigma concerns	6/8	6/7	0/4	11/13	10/14	2/7
Information gap on CARGs	6/8	5/7	2/4	10/15	10/14	0/7
Few perceived benefits	4/8	3/7	0/4	0/13	0/14	0/7
Work commitments	0/8	1/8	2/2	2/15	0/16	1/7
Participants’ recommendations						
What would make men join CARGs						
Better marketing of CARGs	5/8	4/7	3/4	13/15	10/10	7/9
Incentives (e.g. T‐shirts, bicycles, food, income‐generating projects etc)	5/8	6/8	4/4	3/15	13/1	5/13
CARGs should be self‐formed and not facility‐formed	n.a.^a^	n.a.^a^	3/3	6/13	0/16	12/13

CeL, Central‐Level Participants; CoL, Community Leaders; HCW, Healthcare Workers; MinC, Men on ART in CARGs; MoC, Men on ART not in CARGs; WinC, Women on ART in CARGs.

a Question not asked of participants

### 3.1 Benefits of CARGs

All FGD and KII participants acknowledged the benefits of CARGs (Figure 2). Time‐ and cost‐savings due to reduced clinic visit frequency were the most commonly reported benefits. Most male and female CARG members also mentioned psychosocial support as an important benefit, explaining that CARGs provided a supportive community and reduced feelings of isolation. Psychosocial support from peers was also said to improve ART adherence.

HCWs, policymakers and implementers additionally reported that CARGs led to decongested HFs. Providers noted shorter waiting times for other patients and increased ability to prioritize urgent cases. Community leaders recognized the psychosocial support, decongested HFs and reduced HCW workloads.

FGD and KII participants also noted that CARGs facilitated increased openness about HIV, helping to change community attitudes. HCWs reported that CARGs helped to de‐stigmatize HIV, making it easier and more acceptable to be on treatment. Despite these improvements, participants noted that HIV‐related stigma remained a problem in their communities and accounted for some men’s reluctance to join CARGs.

### 3.2 Challenges of CARGs

The overwhelming majority of CARG members, community leaders and HCWs reported no CARG‐related challenges. A few HCWs and community leaders felt that some CARG group leaders lacked the requisite leadership skills and HCWs reported that some CARGs had to be disbanded because of intra‐group conflict. Other potential sources of conflict included irregular CARG meeting attendance by some members and failure by others to contribute toward transportation costs to the HF as expected.

Some HCWs expressed concern that CARGs made it harder for them to monitor patients on a regular basis (Figure 3). They gave examples of CARGs that sent the same person (often the group leader) to the HF instead of rotating this responsibility as expected. This meant that HCWs saw individual CARG members less frequently than planned.

When asked, most participants preferred self‐formed CARGs, that is, groups of patients who propose themselves as a CARG to HF staff. HCWs at some HFs were reportedly assigning patients to CARG groups, rather than letting them self‐form; this approach was generally disfavoured.

### 3.3 Barriers to male participation in CARGs

Fear of HIV‐related stigma and lack of awareness of CARGs were the most commonly reported barriers to male involvement (Table 3). Additionally, some men who were not CARG members perceived few benefits of joining.

#### 3.3.1 Fear of stigma/confidentiality concerns

All participant groups, except women in CARGs, mentioned HIV‐related stigma as the most important barrier to male enrolment in CARGs (Table 3). Despite acknowledging that CARGs reduced stigma in their communities, participants noted that significant stigma remained. Men not in CARGs were particularly concerned that enrolment required them to disclose their HIV status to others and expressed fear of intentional or unintentional disclosure of their HIV status to other community members. Some men who were not CARG members were also apprehensive that they would find themselves in CARGs with extended family members (e.g. in‐laws) or with community members with whom they were not on friendly terms. Others had intentionally chosen to continue to receive their care at HFs not located in their communities as a way to maintain their confidentiality. Still others were concerned about the inclusion of women in CARGs, stating that they were more likely than men to disclose the HIV status of others in the group. These participants expressed preference for men‐only CARGs.

Fear of HIV‐related stigma was deeply entangled with notions of respectability and manhood; this was a substantial concern for men who were not in CARGs. Many feared that if their HIV‐positive status was widely known in their communities they would be treated as “lesser men” and be disrespected. “Being talked about,” “being laughed at” and “being pointed at” were terms used to describe anticipated community reactions. Finally, men were concerned about being excluded from social activities because they were HIV positive. In contrast, several CARG members shared personal experiences of discrimination but noted that the support they had received from other CARG members had helped them cope with such experiences.

#### 3.3.2 Information gap

Lack of awareness of CARGs was the second most common reason given for lack of male engagement (Table 3). Some men who were not CARG members reported that they had never heard of CARGs. They attributed this to work commitments requiring them to be away from their communities for extended periods. Men also noted that women typically had easier access to health information because they patronized HFs more frequently. The few men not in a CARG who had heard of CARGs reported they had insufficient information about them (e.g. eligibility criteria, how to join), attributing this to poor marketing of CARGs in their communities. Men who were not in CARGs also had misconceptions about this model of care. Some thought that CARGs were for women only.

#### 3.3.3 Few perceived benefits

A third reason for non‐participation in CARGs, suggested mostly by male FGD participants, was that some men did not see any personal benefits to CARG membership because they lived close to HFs and thus had easy access to services (Table 3).

### 3.4 Facilitators of male participation in CARGs

We asked men in CARGs why they joined and other participants what they thought would convince more men to join. We also asked both groups for suggestions to increase CARGs' appeal to men. Reasons for joining revolved around the benefits reported earlier. The three most common suggestions given for increasing male engagement in CARGs were: better marketing; provision of monetary and non‐monetary incentives; and increased flexibility in CARG design and implementation (Table 3).

#### 3.4.1 Better marketing of CARGs

Men who were not CARG members frequently stated that they would join CARGs if they knew more about what they are and how they function. This perspective was echoed by other groups, who further suggested that village health workers, wives and men already in CARGs could more effectively encourage men to join. HCWs and programme implementers suggested taking a more proactive approach by going to men’s workplaces, bars and homes to provide information about CARGs. All participant groups were convinced that these actions would help allay men’s stigma concerns and encourage more men to join CARGs.

#### 3.4.2 Provision of incentives (monetary and non‐monetary)

All participant groups reported that provision of monetary and/or non‐monetary support for CARGs would make them more appealing to men. CARG members noted that funds for income‐generating projects, such as poultry‐raising and gardening, could help alleviate the pressure for men to be economically productive. Other suggestions included: food; bicycles to defray transportation costs; bags to carry members’ medications discreetly; and T‐shirts to help advertise CARGs and reduce HIV‐related stigma. HCWs, implementers and community leaders echoed similar sentiments.

#### 3.4.3 More flexibility in CARG design and implementation

Some men expressed preference for men‐only CARGs, opining that women gossip and cannot maintain confidentiality. Many participants also emphasized the importance of self‐forming rather than “assigned” CARGs. They felt this approach built participant ownership, increased the likelihood of amicable groups, and provided at least partial reassurance that co‐members would guard confidentiality.

## 4 Discussion

We examined knowledge and perceptions regarding CARGs in order to understand male engagement in these groups in rural areas. This study is among the first to specifically investigate male engagement in community‐based DSDM; most studies focus on male engagement in ART more generally 29 or on general challenges of DSDM 30, 31.

Perceptions of CARG benefits and disadvantages among study participants were consistent with data from Malawi, South Africa and Mozambique 31, 32, 33, 34, 35, 36. When asked specifically about reasons why men might not participate in CARGs, participants identified fear of HIV‐related stigma, lack of information about CARGs, lack of male‐only groups and few perceived personal benefits of CARG membership. Although similar findings have been reported in other studies 32, 33, 34, we used an explicitly gendered perspective and examined these barriers from men’s viewpoints.

HIV‐related stigma was experienced in very gendered ways, with men indicating that HIV infection made them feel vulnerable *as men*, particularly in their socially valued roles as husbands, fathers and productive community members. These concerns highlight the close‐knit and communal nature of rural life in Zimbabwe, which makes anonymity difficult. Although secrecy often is interpreted as a form of denial that fuels HIV‐related stigma 35, secrecy can also be a form of agency that allows vulnerable individuals to control who has access to sensitive and potentially harmful information about them 36. Similar findings have been reported in studies in rural Uganda and South Africa, among others 35. Gender dynamics were also an undercurrent in a Malawi study where tension between traditional patriarchal household roles and the expected equality of men and women in a CARG was noted as a challenge 31, 32. Our study adds to this emerging literature by suggesting a direct link between stigma, masculinity concerns and low male involvement in community‐based DSDM.

Although study participants noted that CARGs can promote privacy by decreasing HF visit frequency and hence the chance of being “outed” when seen at the HIV clinic by neighbours or community members, and that CARGs can *decrease* stigma by providing mutual support, potentially mitigating the impact of discrimination, HIV‐related stigma—both experienced and perceived—remains deeply entrenched in Zimbabwe’s rural communities. A 2014 study found that 66% of 1905 Zimbabweans living with HIV reported personal experiences of HIV‐related stigma and discrimination 37, despite an intensive 2005 national anti‐stigma campaign 38 and the widespread availability of free ART since 2004. Thus, gender‐specific stigma‐reduction campaigns that speak explicitly to the masculinity concerns of men living with HIV should be prioritized. Community leaders, village health workers, HCWs and men who are open about their HIV‐positive status could be effective allies in such stigma‐reduction campaigns. While men (and women) who strongly desire to conceal their HIV status may prefer not to engage with group treatment models like CARGs, better explaining the potential benefits, encouraging self‐formed groups, and permitting male‐only groups when desired, may encourage CARG enrolment. Additionally, other DSDM that do not require group membership, such as Fast Track Refill and Family Pickup models, should be actively promoted in rural communities.

Limited awareness and knowledge of CARGs was another key barrier to male engagement. This finding confirms that men’s limited access to health information is a persistent challenge. Lack of information about health services has long been cited as a common barrier to men’s healthcare‐utilization behaviour globally 39 and more recently, to low uptake of community‐based DSDM. In a Malawi study, ART patients not enrolled in CAGs also reported little awareness of the model 31, 32, whereas in a South African study of adherence clubs, participants were confused about eligibility criteria, enrolment procedures and referral criteria 33. Better and intensified information and education campaigns, preferably in workplace and entertainment venues, are needed to reach more men and more effectively. This is important since many men not in CARGs expressed strong interest in joining once they had learned more about the model.

Additionally, support for CARG‐related income‐generating activities and provision of material support for CARG members may directly incentivize participation, and may also contribute indirectly, by addressing the connection between masculinity and earned income. Studies of male engagement in HIV peer support groups have found that income‐generating activities may cushion men from HIV‐related stigma 40. A Malawi study found that income‐generating activities, though not part of the formal CARG structure, were spontaneously initiated and highly valued 31, 32. Linking CARGs to local organizations that offer such support may be more feasible and sustainable than requiring individual HFs and/or MoHCC to play this role.

Lack of perceived personal benefits of CARG membership was the final key barrier in our study. Some men who lived close to health facilities saw no need to join CARGs, whereas others valued having direct access to HCWs. CARGs are not for everyone and some men (and women) may prefer alternate DSDM. We therefore recommend that awareness campaigns also educate community members on DSDM options.

## 5 Strengths and limitations

This study adds to the growing literature on male engagement in HIV treatment, providing an in‐depth exploration of the impact of a rapidly expanding DSDM from multiple perspectives – men enrolled and not enrolled in CARGs, women enrolled in CARGs, community members, healthcare providers, policymakers and DSD programme implementers. Most qualitative studies aim to provide description, explanations of and meanings to phenomena in specific situations rather than generalizability of findings. However, data rigour, for example, triangulation of findings from diverse stakeholders, use of both KIIs and FGDs, and application of a constant comparison approach in data analysis helped confirm the consistency and enhance internal validity of our study findings.

Study limitations included purposive sampling of participants from three clinics in two rural districts with two broad age groups (18 to 35 years and 36+) and exclusion of patients who no longer participated in CARGs. Thus, our findings may not reflect the attitudes, beliefs and experiences regarding CARGs in other parts of Zimbabwe, or the perspectives of men who disengaged from CARG participation.

## 6 Conclusions

Men in CARGs clearly articulated the benefit of participation. Addressing barriers to CARG engagement may encourage enrolment and enhance the experiences of other HIV‐positive men, leading to improved satisfaction and possibly to enhanced treatment outcomes. To achieve this, targeted informational, educational and communication programs that clearly explain what CARGs are, how they function and how to join are a priority, as is bringing this information to men in their workplaces and communities. Flexibility with regard to CARG design and support for self‐forming groups rather than “assigned” groups are likely to address some men’s concerns about privacy and confidentiality. Material and in‐kind support may also increase enrolment and retention in CARGs. Some men may prefer alternative DSDM, including those that are facility‐based and/or do not require group participation. Finally, providing diverse male‐friendly services—not just optimized DSDM—will be important to fully engaging men in the HIV response. For example, integrating HIV and non‐HIV men’s health services—including those for mental health, sexual health, primary care and non‐communicable diseases—may further help to reduce stigma and increase male engagement.

The role of stigma in men’s participation in CARGs is paradoxical, as the model can both generate and mitigate stigma. For some men, CARGs provide not only efficiency and convenience, but a safety net and a warm and receptive environment to receive and share psychosocial support and information about coping with HIV and medication adherence. For others, threats to privacy and the resultant perceived and anticipated stigma were significant disadvantages. We did not find that these perceptions varied by age, although our ability to ascertain these differences may have been constrained by the study’s broad age group categories.

Effective gender‐specific and community‐based HIV‐stigma reduction initiatives are likely to improve male engagement in HIV services in general and CARGs in particular. Whether other DSDM, such as facility‐based appointment spacing models, fast track drug pick‐up, family‐based models or facility‐based group models, are less stigmatizing and provide a more confidential environment than CARGs remains to be explored. No one DSDM is likely to meet the needs and preferences of all recipients of HIV care and countries must decide what mix of DSDM is the best fit for their setting 41.

## Competing interests

None of the authors have competing interests.

## Authors’ contributions

TA, CG, PP and MR developed the study concept. TA, LB, CG, GM, JEM, TBM, MM and MR participated in study design and planning. MM led the data collection team. JEM and TBM led the data analysis, with support from EB and other team members. JEM, TBM and MR drafted the manuscript. All authors read and approved the final article.
